# Access to curriculum for students with disabilities at higher education institutions: How does the National University of Lesotho fare?

**DOI:** 10.4102/ajod.v6i0.257

**Published:** 2017-04-28

**Authors:** Paseka A. Mosia, Nareadi Phasha

**Affiliations:** 1 Inclusive Education Department, University of South Africa, South Africa; 2 Educational Foundations Department, National University of Lesotho, Lesotho

## Abstract

**Background:**

Creating access to curricula at institutions of higher education for students with disabilities requires a concerted effort from management and other key stakeholders to identify students’ needs and create opportunities for success.

**Objectives:**

This paper presents the findings of a study which examined students with disabilities’ access to curricula at a higher education institution in Lesotho.

**Method:**

Data for this qualitative study were collected using three methods: in-depth interviews, focus group discussions and document analysis. Eleven students with various types of impairments and 15 academic and non-academic staff members currently working in close proximity to students with disabilities participated in this study.

**Results:**

The findings reveal inconsistencies between the institution’s admission policy of non-discrimination according to disability status and its practices. These inconsistencies are discussed under the following themes: (1) access at admission level, (2) management of disability data, (3) support by the special education unit, (4) teaching strategies, (5) support by lecturers, (6) availability of assistive technology, (7) special concessions and (8) students’ coping mechanisms.

**Conclusion:**

We recommend that a clear policy concerning the support of students with disabilities be developed with the following aims: guide decisions on how disability data should be used, define roles that different university departments must play in facilitating access to curricula for all students, influence suitable development of teaching and learning resources, stimulate research on success and completion rates of students with disabilities and mandate restructuring of programmes that are currently inaccessible to students with disabilities. Key stakeholders, including students with disabilities, disabled persons’ organisations, disability rights activists, and staff should be involved in such policy design.

## Introduction

Debates concerning access to curricula at higher education institutions (HEIs) for minority groups, particularly persons with disabilities, are characterised by a continued tension between merit and equality. The tension continues to exist even though many years of access for non-traditional groups to study at this level of education have been provided (Leach 2013). One of the many issues raised in this debate concerns the relevance of current syllabi to contemporary student populations in the majority world (see Read, Archer and Leathwood (2003), Reay (1998) and Nkoane (2006) for a discussion of this issue).

Despite the breadth of the issues discussed by these authors, one parallel which the discussion around curriculum relevance has with issues faced by persons with disabilities has to do with accommodation. Writing about cultural adaptation of curricula, Nkoane (2006) argues that each student requires a curriculum that speaks to his or her own issues and taps into his or her areas of creativity and strength. Access to education requires that ‘every aspect of schooling, from policy to curriculum to pedagogical elements, to leadership, to ethos and culture … change in order to educate learners within a common framework’ (Terzi 2014:483). With specific reference to individuals with disabilities, there should be adaptations in terms of teaching approaches as well as the modification of the physical arrangement of the classroom, if required (Habulezi & Phasha 2012). In addition, access should involve using a medium of communication that is appropriate and accessible for all learners to facilitate independent interaction with the content.

Within the access discourses, issues of how social institutions create excellent and equitable opportunities for all to benefit take centre stage. Skrtic (1991) argues that

student disability is neither a human pathology nor an objective distinction; it is an organizational pathology, a matter of not fitting the standard programs of the prevailing paradigm of a professional culture. (p. 169)

Skrtic’s (1991) argument places the responsibility for the exclusion of students with disabilities with their institutions. Although an extreme stance, the creation of access to education for students with disabilities and other minority groups is fundamentally an effort to transform institutions to accommodate and support human diversity. Research from the United Kingdom, Turkey and Canada, however, shows that there are often barriers to universities fulfilling this mandate.

Vickerman and Blundell (2010) indicate that the United Kingdom (UK) has good policies but that these policies are not supported by appropriate staff training explaining educators’ responsibilities for making education at HEIs accessible. Equally, universities are rewarded for producing equity plans and there is funding associated with the inclusion of students with disabilities, and yet some students still hide their disabilities to avoid discrimination (Riddell & Weedon 2014). Countries such as the United States of America (USA) have well-known policies and legislation supporting the right to education for students with disabilities at all levels, however, implementing those policies remains a problem. At the level of implementing policy, then, support for students with disabilities has been found lacking.

Furthermore, according to Gelbar et al. (2015), students with disabilities still face challenges such as inaccessible buildings, rigid curricula and negative attitudes of staff and lecturers who lack information on disability issues and allow only minor accommodations which constrain access to education for students with disabilities (Murray, Wren & Keys 2008). Likewise, in Turkey, the needs of students with disabilities are ignored (Arslan-Ari & Inan 2010) mainly because of poor resources and the placement of disability units under the authority departments that do not deal with disability issues. Without an independent budget, services to students with disabilities are negatively affected. Mullins and Preyde’s (2013) study in Canada revealed that despite the country’s policies which require HEIs to eliminate physical barriers and create access, structural barriers remain an obstacle for curriculum access. Sachs and Schreuer (2011) maintain that some institutions pay attention to academic and physical accessibility at the expense of students’ social participation and support. There is a good reason to believe, then, that the inclusion of students with disabilities in HEIs is often ill-executed. Clearly, as these authors note, there is a disjuncture between what policies state, and what occurs, and that the barriers to inclusion and accommodation are not merely practical but also social.

Furthermore, evidence suggests that such policies serve institutional ends which have less to do with the inclusion of students with disabilities, and more to do with funding, and achieving equity goals ‘on paper’. This appears to be the case even in high-income contexts which are not characterised by the constraints on resources found in low-income settings. In the latter context, it is perhaps even more likely that HEIs would lack the requisite resources, skills, and capacity to create, or enact, such policy.

Indeed, research on access practices at HEIs in Lesotho is limited. An extensive literature search revealed only two studies. A survey by the Lesotho Council on Higher Education (CHE) (2012) highlights that many buildings in tertiary institutions in Lesotho are not accessible for students with disabilities, one of the root causes of low participation of these students. Specifically, in the case of the National University of Lesotho (NUL), there is a failure to identify all students with disabilities, especially those with physical impairments (CHE 2012). This survey provides no further details than the basic challenges of identification and low participation of students with disabilities (CHE 2012). The survey does not deal with challenges related to curriculum access for disabled students. In the second, and only other study, Matlosa and Matobo (2007) investigate access constraints faced by the visually and hearing impaired students at HEIs in Lesotho. Their study shows that access for students with visual impairments to science-related programmes is constrained by Mathematics and Statistics requirements, insufficient resources and lecturers’ lack of understanding about the students’ disability and support needs. This study excluded students with other forms of disabilities. An understanding of a wide variety of disabilities is necessary for ensuring curriculum access of the group in question at HEIs. Importantly, hearing from students with disabilities will offer them an opportunity to make contributions on matters that affect them. As an attempt to close the gap, the present study addressed the following questions:

- How accessible are higher institution’s programmes to students with disabilities?
- What practices are in place to facilitate access for students with disabilities, and what challenges – if any – face students with disabilities in their attempts to achieve full and effective participation at the university?

## Conceptual and theoretical framework

In the present paper, we are concerned with access to HEIs. Thinking about access to curricula at tertiary level entails a consideration of equality, equity and justice. These terms attract multiple and polarised interpretations hence the need to establish their meanings for this study. Walker (2003:169) observes that though formal university education makes a positive contribution to people’s lives, ‘it produces justice and injustice, equity and inequity’ if left unmodified to the needs of different groups. Therefore, there is a need to transform the education systems to ensure respect for human rights and to certify the attainment of social justice (Dyson 1999). In the sections which follow here, we consider the equality, social justice, and distributive justice, examining each for their relevance to our study. We focus on Sen’s (1979, 1985, 1999) conception of access, as conceived of in his capabilities approach, supported by a distributive justice framework. We suggest that these two conceptual frames allow for a thinking through of access issues encountered by students with disabilities in HEIs which is particularly useful.

### Equality, social justice and equity

In describing equality, Terzi (2014:484) states that social and institutional activities should assure ‘equal consideration to all’ and provide ‘equal entitlement of every child to education, while acknowledging and respecting individual differences’.

Equality of opportunity in education, in the past, was premised on the idea that – given access, or exposure, to a given curriculum, individuals from any background, might be expected to stand to reap the same gains. The premise, naïve as it was, was that, if educational opportunities were made more widely available to individuals from previously marginalised groups, then those individuals might be expected to achieve just as well, perhaps, as individuals from groups long included in such opportunities. When this conception of equality of opportunity paled in popularity because of its inherent flaws (access and exposure are necessary, but not sufficient, conditions for equality of opportunity), it was followed by another, equally problematic idea: that students with different capacities, should be given ‘equal opportunity’ by being separated and given tasks suitable to their perceived capacity and proposed prospects (Coleman 1968).

Contemporary definitions of equality of educational opportunity, however, now attend to the multiple ways in which histories of inequality and lack of access to education, minority status and social disenfranchisement might impact on individuals’ capacity to enjoy an equal chance to thrive in an educational setting, and so incorporate an idea about participation, and not merely inclusion.

However, given the recognised limitations of the idea of equal opportunity to redress past inequalities, the concept of social justice has come to the fore in higher education access debates. This stems from the recognition that inaccessibility, or failure to adequately profit from tertiary education, arises from complex web of social injustices related to students’ socio-economic contexts, race and geopolitical position. Usefully elaborated by Sen (1979, 1985, 1999), the capabilities approach to higher education shows how any attempt to create social justice in educational settings must incorporate both a consideration of what a given individual can do and the individuals’ ability to enjoy their abilities in the context of opportunities (Wilson-Strydom 2011). The first concerns Sen termed functionings – achieved outcomes, the things that a person is able to be or to do, and the second, capabilities – which combines the idea of functionings with equality of opportunity. Functionings are ‘actions and states that people want to achieve and engage in’ (Terzi 2014:485).

At tertiary level, functionings relate to the ability of students with disabilities to take part in the curricula activities without barriers and achieve desired outcomes (Wilson-Strydom 2011). Conversely, capabilities are ‘the genuine, effective opportunities that people have to achieve valued functionings’ (Terzi 2014:485). Capabilities are enhanced if students have more educational opportunities than disadvantages and marginalisation (Wilson-Strydom 2011). Capabilities, then, are the freedom a person has to enjoy valuable functionings (Alkire & Deneulin 2009; Deneulin, Nebel & Sagovsky 2006; Sen 1979, 1999). The capabilities approach, as Wilson-Strydom (2011) points out, entails a clarion call to universities to achieve access with

both [*the*] redistribution of resources and opportunities and recognition and equal valuing of diversity along intersecting axes of gender, social class, race, ethnicity, disability, age and so on. It thus integrates distributional, recognitional and process elements of justice. (p. 411)

As Wilson-Strydom (2011) writes,

The capability approach argues that in a just world social structures or social organisations should expand people’s capabilities – their freedom to achieve what they value doing and being. Capabilities (opportunity freedoms) and functionings (achievements) are influenced by individual circumstances, relationships with others, social conditions and contexts which create spaces for opportunities to be realised. (p. 412)

### Distributive justice

The capability approach, and an expanded definition of social justice, implies that individual differences should be catered to in educational settings. In order to achieve such recognition of individual differences in life histories and goals, while not re-perpetuating the faults of early conceptions of equal opportunity, necessitates a consideration of the idea of distributive justice.

Salmi and Bassett (2014) ascribe the development of the distributive justice model to the work of John Rawls (1985), Amartya Sen (1985), and Ronald Dworkin (1981) among others. There are several perspectives of social justice among which is the distributive component (Gale & Tranter 2011); hence Singh’s (2011) claim that the meanings and uses of social justice are becoming stretched in different directions. Some distributive justice perspectives denote the equality and justice ideas referred to above which focus on fairness and sameness. However, Mckee (1981) argues that distributive justice calls for all perspectives of justice to operate at once. That is, individuals should be afforded the freedom of making choices and be compensated for their disadvantage through positive discrimination (distributive) (Gewirtz 1998). Individuals’ rights should be protected and they should be exposed to the same conditions or services and punished individually for a violation of rights (retributive). In addition, all must have choices to achieve their potential through processes that promote the interests of minority groups (recognitive) (Gale & Tranter 2011).

According to Rawls (1971:6), justice is brought by ‘the way in which the major social institutions distribute fundamental rights and duties and determine the division of advantages from social cooperation’. That means that people’s natural features should neither give them superiority nor make them worse off; impairments are just natural human attributes but the way social institutions distribute goods and services may result in just or unjust practices for individuals with impairments (Rawls 1971). Walker (2003:172) adds that ‘our preferences and choices are shaped and informed or deformed by society and public policies’. Our argument resonates with Dworkin’s (1981:302) assertion that individuals with impairments face their lives ‘with what we concede to be fewer resources, just on that account, than others do. This justifies compensation, under the scheme devoted to equality of resources’. Therefore, unless there are any concrete efforts by tertiary institutions to create meaningful opportunities for people with disabilities, access to curricula would have been denied.

Distributive justice obliges HEIs to have (1) a modified admission requirement to cater for the diverse pre-tertiary education context of minority groups and (2) curricula reformed and support enabled to safeguard those admitted to have ‘practical and socially meaningful educational success’ (Waetjen 2006:205). Sen’s (1985) notion of access, as conceived of in the capabilities approach, also suggests that disadvantage should be compensated.

## Research design and methodology

This qualitative study adopted a single case study design. The design offers the benefit of investigating a single unit intensively (Yin 1994). Taken as a whole, qualitative research is ‘inductive, subjective, and contextual’ in nature, and offers an opportunity to capture the unique experiences and beliefs of participants in their interaction with their context (Morgan 2014:47). In the context of the present study, exploratory as it is, such an approach affords us the opportunity to examine the participants’ accounts in depth, and allows for a nuanced understanding of their experiences. In exploratory research, such nuancing is imperative, as it cleaves open new areas of necessary inquiry to direct future work.

### Research location

The study took place at one of the HEIs in Lesotho. The institution was established in 1945 as a Catholic institution affiliated to the University of South Africa (NUL Calendar 2006/7). The university admits 43.9% of Lesotho’s undergraduate student population and 89.4% of postgraduate students. The total number of students enrolled at the institutions was 11 363 in 2011/2012 (CHE 2012:9).

### Sampling and participants

Participants were identified by means of a purposive sampling technique – snowball sampling. Purposive sampling affords an opportunity to reach ‘rich information cases’ (Patton 1990). Snowballing, in particular, is important for a hard to reach population (Corbetta 2003), such as students with disabilities. The first three students were referred to the study by a Special Education Needs Assistant (SENA). Thereafter, following an interview, the participating students were asked to invite their peers with disabilities who may be willing to share their views about access to curricula. The criteria for selecting students included: being 18 years and/or above, registration in any field of study, willingness to participate in either in-depth interview or focus group discussions, and having impairment. Gender, ethnicity and year of study did not form part of the selection criteria. We ended with a sample consisting of 11 students with disabilities.

The students and the SENA were of assistance in obtaining a sample consisting of 15 staff members at the institution whose responsibilities and the services they offer permit them closer contact with students with disabilities such as those working in the academic (10) and support units (5). The referees provided names and contact details of staff members who they thought might be interested in participating in the study. The first contacts with staff members were made by email, and then followed by a meeting to discuss the purpose of the study and to finalise the interview date. The selection criteria for staff included: having taught a disabled student or provided a support service to such a student (see Tables 1 and 2 below for detail description of participants, pseudonyms and titles of the staff members).

**TABLE 1:** Students with disabilities.

Name	Disability category	Gender	Age	Programme	Year level
Thomas	Blind	Male	29	Bachelor of Education	3
Keletso	Partially sighted	Female	22	BA Social Work	4
Thabo	Partially sighted	Male	24	Bachelor of Education	1
Lerato	Physical disability	Female	27	BSc Consumer Science	2
Raphael	Physical disability	Male	27	BA Social Work	3
Katleho	Partially sighted	Male	29	Diploma in Mass Communication	3
Norma	Blind	Female	47	Postgraduate Diploma in Education	1
Karabo	Physical disability	Male	33	BA in Adult Education	1
Lineo	Physical disability	Female	22	Diploma in Business Management	2
Motse	Physical disability	Male	29	Diploma in Adult Education	3
Thetso	Deaf	Female	37	Diploma in Pastoral Care	1

**TABLE 2:** Staff participants.

Description of participants	Number of participants	Male	Female
FED lecturers	2	1	1
FOH lecturers	1	1	0
Faculty of Social Sciences lecturers: Sociology, Social Work, and Business Administration	4	2	2
Faculty of Science lecturers: Consumer Science	1	0	1
IEMS lecturers	2	1	1
Library staff	1	1	0
Welfare officer	1	1	0
Counsellor	1	0	1
Admissions officer	1	0	1
SENA	1	1	0

**Total**	**15**	**8**	**7**

FED, Faculty of Education; FOH, Faculty of Humanities; IEMS, Institute of Extramural Studies; SENA, Special Education Needs Assistant.

At the time of data collection, the university did not have comprehensive records of all registered students with disabilities. Therefore, this study cannot calculate what percentage the 11 student participants represent of the total number of students with disabilities enrolled at the university at the time of data collection.

### Data collection and analysis methods

The study collected data by means of three methods: individual in-depth interviews, focus group discussions and document analysis.

### In-depth interviews

Interviews were semi-structured; guided by statements phrased into questions but flexible enough to allow research participants to raise issues pertinent for the study (Hugh-Jones 2010), and to express themselves in their own individual ways and at their own pace

In-depth interviews lasted between 60 and 90 min. They took place at the university in a room allocated for this purpose. The first author facilitated all the interviews using English or a combination of English and participants’ home language, which is Sesotho. They were scheduled during participants’ free time when they did not have classes and/or during weekends. All the interviews were tape recorded with participants’ permission following thorough explanation of research ethics including the principle of non-payment for participation.

This form of data collection gave participants a platform to have their voices heard regarding, in particular, policies and practices in relation to access. According to Morgan (2014), semi-structured interviews permit students to respond to questions freely while giving the researcher ample opportunity to gather participants’ additional insight on the topic. A sign language interpreter was involved during the interview with a deaf student. The interpreter was involved with the permission of the deaf student and a thorough explanation of research ethics, which were also followed by a written declaration. The interviews with each participant were conducted face-to-face.

### Focus group discussions

Following in-depth interviews with all students, five students who were studying full time were invited to participate in a focus group discussion. Of these, two are living with physical disabilities and three are living with visual impairments. These focus group discussions enabled us to follow up on issues that had arisen during the in-depth interviews and analysis of documents. Participants were also able to share some information they withhold and/or exaggerated during individual interviews. Participants had an opportunity to critique and discuss each other’s statements (Gibson & Riley 2010). Overall, focus discussions allowed us to capture a wide variety of views within a short space of time.

### Documents

To complement the interview data, documents were analysed. These included university brochures, which are usually given to new applicants by the admissions department (available on the institution’s website), reports, minutes of meetings, and internal Memoranda (MEMO) provided by the SENA and those filed under Special Education Needs folder. Permission to use the institution documents such as MEMOs and Brochures was sought from the Registrar and relevant offices including the office of the Dean, Faculty of Education and Admission office.

### Data analysis

Interpretative phenomenological analysis (IPA) was used as a data analysis approach to examine participants’ narratives of their unique experiences (Smith 2011). IPA provides an insider’s perspective of the subject and uses individual cases as the basis for explaining broader social issues (Larkin, Watts & Clifton 2006). Data were coded to discern ‘patterns that point to a theoretical understanding of social life’ (Babbie 2014:409). Coding involved scrutinising each individual case very closely and searching for similar or different patterns across cases (Smith 2011) to come up with themes and sub-themes. This was followed by establishing relationships between sub-themes and themes to enable us to understand curriculum access for students with disabilities at the HEI.

As in any qualitative study, the process began in the field to ensure that subsequent data collection stayed focused. Following each interview, the researchers listened to the recorded data to identify tentative themes and sub-themes, which were refined as soon as transcribed data became available. The process also involved establishing links between themes and sub-themes and was carried out until data saturation – that is, until the transcripts were no longer revealing information which made a novel contribution to the researchers’ understanding of the topic, as revealed by the themes and sub-themes.

## Results

The findings of the study are presented under the following themes: (1) access at admission level, (2) the use of students’ disability data, (3) support by special education unit, (4) teaching strategies, (5) support by lecturers, (6) availability of technology to facilitate curriculum access, (7) special concessions and (8) students’ coping mechanisms. In presenting the findings, participants’ words are quoted verbatim.

### Access at admission level

According to the institution calendar (2006/2007:11), admission is open to all students irrespective of their race, religion, gender or disability status. Staff members indicated that programmes and courses are open to all students and they did not know of any student who had been refused admission on the basis of disabilities. However, a staff member did reveal that students with disabilities were more likely to enrol in humanities courses, as opposed to those in the science or business faculties. As a SENA stated:

‘The programmes they [*students with disability*] register for are usually in the Humanities. They do not delve into the sciences and other programmes.’ (Participant 11, Male, Non-academic Staff)

These statements seem to imply that – although on paper students with disabilities had equal access to all faculties – there might be institutional barriers to their ability to choose freely from different courses. Furthermore, as a sociology lecturer explained, there were accessibility issues which were thought responsible for some students with disabilities not to enrol:

‘Students with visual disabilities cannot do Economics because they would be required to be in front of computers manipulating data and all that.’ (Participant 24, Female, Academic Staff)

Comments from the Admission Officer suggested that the accessibility issue might also be one of staff perceptions of the capabilities of students with disabilities:

‘I remember one faculty was about to reject or, in fact, it rejected them. It was stated that students with visual impairment were not admissible in the Faculty of Law while it had previously admitted such learners who studied until they finished their Law degrees. I think social sciences previously rejected them indicating that they have not yet secured equipment for their needs.’ (Participant 25, Female, Non-academic Staff)

The students with disabilities also shared their experiences, which contrasted with the university admission statement, and reflected various ways in which they were discouraged to follow particular areas of specialisation. Karabo, a student with cerebral palsy, whose first choice of study was Mass Communication, noted:

‘I was told there were no resources for admitting me to do my first year at Diploma level [*in Mass Communication*]. I was not admitted and then I went to the Special Education Unit of the Ministry of Education and Training because they mentioned that if a student with disability meets the requirements, he or she [*sic*] must be given a chance. So admission criteria are problematic because they will admit a person who falls within their scope of education. The question is, which university should I go to if my needs are not catered for in this institution?’ (Participant 8, Male, Student)

A deaf student, Thetso, who wanted to do a Law qualification stated:

‘The lecturer told me that I would not be able to cope being here at school because you would have to sit in front and lip-read the lecturers.’ (Participant 26, Female, Student)

Citing lack of facilities as the reason for discouraging students to follow particular areas of specialisations demonstrates the institution’s rigidity to accommodate students whose mode of function is different from the norm. It also demonstrates the lecturer’s misunderstanding of disability. The lecturer does not understand that lip reading does not only require sitting the deaf student in the front, but it also requires lecturer’s training in several aspects including talking position and pace, facial expressions and mouth shaping as training the deaf student in concentration and discerning words from the mouth shape of the lecturer.

Karabo further noted:

‘When I first came to the Institute of Extra Mural Studies [*IEMS*] here, Adult Education was not my choice course of study. The then head of department explained to me what adult education is and maybe they saw that I would not be able to do Mass Communication as I had applied to do Mass Comm.’ (Participant 8, Male, Student)

The student felt that he was channelled to a particular field of study, despite his uncertainty about such a premise. He might have been channelled to Adult Education because of speech complication, which might have been revealed during his interaction with the head of the department.

The information obtained from documents attest to the students’ experiences. For example, paragraph one of a memorandum from the Dean of Student Affairs to interim Head-Special Education, dated 8th July 2009, revealed tendencies to discourage students from following particular areas of specialisation because of their disabilities. It reads:

I confirm that ever since her enrolment at the University, Mary [*pseudonym*] has experienced hearing problems leading to a situation where she does not do well in her academic pursuits. When she joined the institution in the academic year 2004/2005 she was doing Law and she was advised to change programmes when she could not make it because we thought she failed on the grounds of hearing difficulty as she would not freely join others in legal arguments and discussions.

The experiences above reflect gaps in terms of university policies and practices. Admission policy claims to be open to all yet in practice students with disabilities are restricted from following particular programmes for reasons such as lack of resources and the perception that they will not cope.

### Use of students’ disability data

Students are encouraged in the application form to declare their disability status and their support needs as the institution ‘is committed to respond to the needs of students with disabilities’ (NUL 2015:2). However, such information is not used to improve curriculum access for students with disabilities. Although the Admissions Officer claimed that information about students’ disabilities is captured on the system and the list of all registered students is submitted to the faculties, she also noted, ‘I have never seen that list sent out with the disability information’ (Participant 25, Female, Non-academic Staff). A lecturer at the institution’s IEMS concurs, claiming, ‘I’ve never had cases where somebody is indicating clearly that as an applicant she or he has some disabilities’. (Participant 19, Male, Academic Staff)

It appears that information about a student’s disability status is not send out to faculties. Students’ disabilities are identified accidentally as evident in the MEMO from SENA to the Dean of the Faculty of Education (23/07/2015). Nevertheless, the institution admits qualifying deaf students and their hearing friends for ease of interpretation of sign language:

It came to my attention through rumours that the University has admitted a student who is deaf, and this morning I actually met two ladies using sign language in the corridors and stopped to ask a few questions out of interest … a student … has been admitted in diploma in Pastoral Counselling Programme with her friend so that her friend would help with interpretations.

Failure of students to make their disability known and to request the necessary accommodation causes delays in accessing curricula, as in the case of a deaf student who secured the services of a sign language interpreter in the seventh week of the first semester. As a consequence, the necessary modifications and adjustments of the learning environment are not implemented. Lerato, a student with a physical disability, laments the fact that when timetables are set there is no consideration of her disability; classes take place in buildings that are far apart, and as a result she misses the first part of the lecture. She explains:

‘I sometimes have problems because you find that I have 8 o’clock class in the Faculty and 9 o’clock class at the BTM, so it means I have to walk long distance. When I arrive in class I’d find that the lecturer has covered much and I’ve missed so many things between ten minutes. I’ll find some lecturers covering but as for others in the Faculty there’s the other who was teaching us and I had to explain to her, and she had to wait for me to arrive.’ (Participant 4, Female, Student)

Raphael adds:

‘I don’t know if they are aware I’m there, they just don’t care. I mean, for example, some of my classes like I said 30% of my classes, which I’m forced like in the 1st floor at BTM.’ (Participant 5, Male, Student)

Some lecturers were experienced as being insensitive to students with visual impairments, thus making them feel excluded from the learning process. For example, Thomas claims he always has to explain himself to lecturers:

‘I think in class, like I said, lecturers do not know anything about me … when they teach, maybe on the board they point and just saying this, “you see this and that”, and to me this and that is not clear. I don’t know what is that…many times I meet lecturers who doesn’t know anything about me.’ (Participant 1, Male, Student)

Lack of support for some students with disabilities was credited with their withdrawal from the university, as in the case of a student who could not cope with the demands of attending classes in venues that were far apart. The Faculty of Humanities (FOH) lecturer recounts:

‘I have a particular [*case*] of a student who enrolled for our programme in the past two to three years. She was having physical impairment…her type of disability was such that it was hard for her to attend some of the classes in the halls that were in the upper levels. That was one thing, the second thing was, sometimes the rooms were so separated that it would allow only students who are said to be able to move from one hall to the other [*timeously*]. Physically they had to run from one hall to another. So she was not able to meet this kind of demand and, as a result, she was disadvantaged.’ (Participant 14, Male, Academic Staff)

It can be suggested that the physical infrastructure at the HEI is not friendly to students with physical disabilities. Some may have dropped out from the institution because of the failure to attend classes that are in the upper levels and the movement from one hall to the other.

It is not impossible that the student and staff accounts of the barriers to education, and causes for dropping out, experienced by students with disabilities are biased, and – indeed – in this sort of research, which deals with emotive and controversial issues, it is impossible to disentangle perception from fact – and it is not our goal. However, what these extracts do evidence is a lack of felt support among students with disabilities, and a perception, among the staff surveyed, that there was a failure to adequately make the requisite accommodations which these students required.

### Support by Special Education Unit

Since 1999/2000, the HEI created a position for a SENA to support students with disabilities. However, the support tends to be inadequate and geared towards one category of disability, namely visual impairment. This was clear in this assistant’s description of his work:

‘We primarily offer braille transcription. That is our main area of activity. We transcribe materials into braille and we can also transcribe from braille to normal text, so it’s just braille in essence.’ (Participant 11, Male, Non-academic Staff)

The SENA also administers tests and examinations written by students with visual impairment in a specially designed computer laboratory for individuals with visual impairments and manages computers installed with Jobs Access with Speech (JAWS). The library has the same hardware services for students with visual impairments. However, these services may not be known to all students, possibly because of poor management of disability data. As a consequence, students may only access these services some months after the commencement of the academic year. For example, Norma, a student with a visual impairment, only became aware of the availability of the JAWS computer laboratory on the day of the examination. She did not receive training on how to use the computers. She explains:

‘I was told that I was also writing while I had not oriented myself with the computers I was going to use for taking the test … . I should have practised with the computers before I could use them for writing a test.’ (Participant 7, Female, Student)

It was also evident that partially sighted students struggle with Internet access, software compatibility and a lack of vision enhancement facilities. The operating systems of the computers both in the library and the unit were outdated and incompatible with JAWS. In the Special Education Service Unit (SESU), they use JAWS 13 with Windows XP. Lerato explains:

‘When we were in first year we struggled with internet. Most of the time our assignments, because we can’t access books, we have to use internet to access books. So most of the time there won’t be internet in the office.’ (Participant 4, Female, Student)

Seemingly, the institution does not have user-friendly study materials and resources including brailed books and books written in large print to facilitate access to curriculum by students with partial sight as a student uses the Internet. This challenge, though it places more burden on disabled students, is not unique to students with disabilities but affects all students. However, as Thomas notes, the issues faced by students with disabilities are perhaps more complex, and more in number, than those faced by the rest of the student body:

‘[*Computer software*] cannot read some, so many things, it needs to be eh installed with OS Windows 08 too, so that it can be easily accessible. For instance when I’m getting into internet I can’t be able to read so many things there. It becomes slow and it takes long, a lot of time to access things. It [*computer*] is totally not compatible [*with the installed windows software*]. I write exams and tests with my own laptop. I am totally not using those ones. Yes, and I think they are useless.’ (Participant 1, Male, Student)

The challenges brought by computers with outdated software are confirmed in a MEMO from SENA to Head-Computer Services Unit (23/03/2015), which reads:

It has come to the realisation of the office (Special Education Needs) that the computers and related services currently available at the ADC Computer lab have become incredibly slow. Inevitably, students’ academic work is frequently held back by the dwindling speeds of this ICT infrastructure. I, therefore, humbly, ask that students…be allowed and assisted to access internet services provided through the ADC Computer Lab using their personal laptop computers as they will outperform the two desktop computers and, in…[*name omitted*]’s case, have assistive software (JAWS) already installed.

### Teaching strategies

Lectures at the institution are offered mainly face-to-face. Students are therefore expected to take notes of the lecturer’s verbal or written presentation on the board. This method is not accommodative of the learning styles and pace of students with disabilities who may experience challenges when taking notes of the materials presented. Since projectors are not used, students with visual impairment (partially sighted) struggle to capture notes if the lecturer’s handwriting is not readable. The problem is worsened by the fact that there are no prescribed books for most courses and that available textbooks are not transcribed in alternative format such as large print to facilitate access for students with disabilities such as partial sightedness. The students’ frustrations are expressed in the following excerpts:

Katleho:

‘Sometimes you would find that I would not be writing because questions would be written on the board and I explain that I can’t, I would not be using the pace of other students. They normally write faster than me, and when the lecturer is through with one side of the board, she would wipe off and write more questions. In such cases I would fail and I remember one of these lecturers would set another paper for me while another would read the questions for me after writing them on the board. There are cases where I was promised another test which never came.’ (Participant 6, Male, Student)

Karabo explains that accommodations appear to be left to the lecturer’s discretion, meaning that, at times, students with disabilities are left behind due to teacher’s failure to recognise and cater to their needs. He recounts:

‘If a lecturer does not cater for my speed in taking notes, it means I am left behind. Either I stop writing and listen or when I write I would miss certain parts of the lesson.’ (Participant 8, Male, Student)

### Lecturer support

Some lecturers’ lack of support poses a challenge to curriculum access for students with disabilities. In particular, lecturers tend to respond negatively when students with disabilities seek support by asking for clarity regarding previous lessons. This was clearly articulated by Motse:

‘ … a lecturer would say something and I would struggle to catch it as fast as other learners. In the next class when one asks questions concerning the previous lesson, some lecturers ask why you did not ask during that lesson. Sometimes you ask the next day that you did not understand much of the previous lesson, and you find that the lecturer is not eager to get back to what was taught in that lesson whereas other students would have understood well.’ (Participant 10 Male, Student)

Also Keletso, who received the notes from a statistics lecturer in her first year at the university, says:

‘Others you’ll have to remind them every minute that after this class I have to have this and then sometimes they don’t prepare what they have to prepare.’ (Participant 2, Female, Student)

Similarly, Katleho mentioned that some lecturers tried to support him even though he could see that they were forcing themselves into working with him:

‘Some of them would say that they need to be trained before dealing with my needs but I would assure them that my challenges were not severe. There are cases where I was promised another test that never came. It wasn’t good at all, in fact last year I had to supplement courses.’ (Participant 6, Male Student)

Clearly, students with disabilities at the HEI have to make other arrangements for their studies because lecturers’ support is not always guaranteed. Even when attempts to provide support to the students were made, it was described as inadequate. An FOH lecturer states:

‘I think my experience is that eh, the support that we gave was not sufficient in a number of ways in that, either the lecturers were not aware; by awareness I don’t mean seeing that they are around, but that they are around and they needed a special type of attention because of their impairments or disability.’ (Participant 14, Male, Academic Staff)

Sometimes, it took the intervention of Special Needs Unit for students with disabilities to get support from lecturers. The memorandum below from the SENA to Dean, Faculty of Education dated 12th February 2015 attests:

I hereby kindly confirm that Mr. Thomas will be sitting for his T323 examination this afternoon at 14:00. Consequently, he will be unable to attend his lectures, ELX3034 and ELG3044 scheduled for 14:10 and 15:10 respectively. Considering the importance of both the examination and the lectures to his academic and professional development, I humbly request that today’s lecture notes and complementary course material be prepared and provided to him by the concerned lecturers.

A lack of adequate support for students with disabilities could be explained by the lecturers’ limited understanding of disability and the needs of such individuals, a problem which can also be traced to management of disability data by the university.

### Availability of assistive technology to facilitate curriculum access

Students with disabilities rely on assistive technology to enhance their learning and access curricula. Such equipment is provided by the institution but may not be accessed by all students who need them, for various reasons. As many of the students cannot afford sophisticated and advanced technologies, they resort to those that require augmentation. They often take a long time to transcribe material and sometimes omit important information from the lecture. Katleho explains:

‘I got a voice recorder, but what I can tell is that when you are about to revise you need to take time because you need to listen to all, let’s say there is a two hours lecture and we have a total of fifteen weeks per semester …. It is a waste of time because a sighted person can go directly to sections he wants in his notes.’ (Participant 6, Male, Student)

Similarly, Norma states:

‘I use my laptop to record the lessons and let me tell you the disadvantage, the disadvantage of recording a lecturer for two hours is that where they laugh, cracking jokes, it records everything…this is different from someone who was using pen and paper for copying only important points of the lesson.’ (Participant 7, Female, Student)

Also, Karabo reports:

‘I have bought a laptop, which enables me to record lecturers [*but*] I don’t have anything to give the lecturer to amplify the voice so that when he moves around [*I*] can still record the voice well. Therefore, the laptop still fails to help me capture all information shared by lecturers. To date, I still don’t know how to overcome this problem.’ (Participant 8, Male, Student)

Thus the inadequacies of students’ technologies compromise their learning. It would benefit students if lecturers made teaching resources, such as notes, accessible to students and were cognisant of students’ use of technology which requires that lecturers project their voices appropriately.

### Special concessions

A generally applied accommodation by the institution is time extension during tests and examinations. For students with visual impairments, time accommodation is consistently 15 and 30 min for tests and examinations, respectively, as per the discretion SENA. Their examination takes place in the laboratory under the supervision of the SENA. However, for the partially sighted, whose tests and examinations are written elsewhere, accommodations differ. Keletso says her time is extended by 15 to 20 minutes for tests, but the time usually just covers time delays in giving her a question paper. She notes:

‘ … sometimes they even forget to set the question paper. I’m delayed for my tests sometimes, and start after time, all those things.’ (Participant 2, Female, Student)

However, Katleho’s tests are written without time extension. He notes:

‘ … as classes start late, we would be writing from 5 to 7 p.m. and at 7 p.m. lecturers expect everyone to be done without excuses. You should understand that I’m writing with a pen, I was no longer used to pen and paper writing. My eyes normally get tired while writing, so when time is up some would just take papers. she would be expecting everyone to give out their answering scripts. Though she may not be speaking to me alone but when she picks her bag and says she is going, you have no choice but to hand in the paper.’ (Participant 6, Male, Student)

As students with difficulties with fine motor skills, Karabo and Motse are allowed an extra hour for each 3-h examination. This differs from 30 min allowed for students with visual impairments. It is not clear how the distinction in time accommodation is decided because staff and students have contradictory views and expectations about it.

Karabo recounts:

‘ … the doctor recommended that I should be given an hour’s extra time for every three hours and I manage to write within that time. Most of our examinations take three hours and I normally finish in three hours forty-five minutes, three hours thirty minutes depending on how demanding a paper would be. Tests are normally not long; it is normally one or two questions written in one hour. Lecturers do allow me to write beyond that hour.’ (Participant 8, Male, Student)

The university rules seem to be relaxed when it comes to Karabo’s tests and examinations supervision. The flexibility is confirmed by the IEMS Lecturer 2:

‘ … an hour or so but we usually have someone to be there to wait for him until he has finished writing.’ (Participant 22, Female, Academic Staff)

This is inconsistent with the conditions provided for Katleho who also studies at IEMS, as his statement above reveals.

Students with visual impairments, on the other hand, feel that the duration of tests and examinations is limited and their time accommodation might have been decided on according to generalised accommodation guidelines for all disabilities. This is a problem as different impairments require different accommodations (for instance, it might take a student with a visual impairment longer to read a braille text than it would take a deaf student to read it). Thomas explains:

‘I’m reading braille, I’m reading with hands and, at the same time, I’m using hands writing on a laptop, typing. Yes, to type is not that easy for me, and writing tests and, at the same time, touching braille. The time is limited because the added time is only thirty minutes.’ (Participant 1, Male, Student)

Norma also states:

‘ … if a normally sighted person is reading a page, when the same page is brailed, not embossed as embossing is like translating, a normal printed page turns into three or four pages when brailed, it makes a pile. Feeling the braille is much work and is better if the questions are in a soft copy.’ (Participant 7, Female, Student)

The two students with visual impairments feel that their time allowance in both tests and examinations is insufficient for their mode of learning. Norma even suggests getting question papers as e-texts instead.

### Students’ coping mechanisms

In the absence of support from the university or when it is limited, students with disabilities come to depend on their peers to access curricula. In particular, they rely on peers for notes, finding library resources and discussions.

Thomas notes with regard to library resources:

‘When I write an assignment, I have to just check my references or bibliography on the internet. No books, unless someone could help me to find a book in the library. [*Other students*] just read for me.’ (Participant 1, Male, Student)

A staff participant from the library confirmed:

‘Actually, in most cases they have their friends but in the case where there is no one, we go there and identify books and then bring books into that room.’ (Participant 12, Male, Non-academic Staff)

Students also depend on the discussion groups with their peers. Through those discussions they are able to access the content they may have missed during the lectures.**** Thomas explains:

‘I’m coping through discussions with my classmates, only discussion helps. I’m not independent because when they are busy with their works, or rather when they would like to read individually, I’ll have to wait for them to come and discuss, and at that time there’d be nothing I could do. I just have to rest and wait for them to come.’ (Participant 1, Male, Student)

Karabo shares the following:

‘I learn some of the content during group discussion but it depends how far time has advanced at the time of discussion. At times you only understand something when discussing for the exam, it is useless because you only memorise it for the exam and did not get it during normal lessons.’ (Participant 8, Male, Student)

Notes of fellow students are not always helpful. Motse states:

‘At times you look through notes of students from your region, only to find that the notes miss a section you wanted. Students copy notes to suit their needs rather than capture everything. You find that students are not able to explain good enough for you to understand.’ (Participant 10, Male, Student)

Insufficient support to students with disabilities leaves them vulnerable to their peers’ misrepresentation of lecturers’ lessons given that there are no prescribed books for all student participants’ courses. Thetso indicates with regard to difficult-to-negotiate sign language interpretation services, which leave her reliant on a peer to assist her with her work:

‘One of the students is helping me, assisting me with the interpretation services.’ (Participant 26, Female, Student)

## Discussion

Findings of this study suggest that there is a disjuncture between students’ experiences of university accommodation practices, and current university policies regarding inclusion and accommodation. For example, the university calendar (2006/2007:11) states, ‘There are no racial, religious, gender or handicap barriers to admission’, but staff noted that students with visual impairments are not admitted in programmes that require Mathematics and Statistics as prerequisites. Students’ experiences confirm observations by staff that some programmes have restrictions that limit the students’ choices. This provides evidence of limitations which deny access to some programmes offered by HEI and, as Skrtic (1991) asserts, inflexible programmes are discriminatory to students with disabilities. In this regard, Read et al. (2003) argue that education which does not accommodate student diversity perpetuates inequality in society. These restrictions also violate the Capabilities Approach’s virtue of choice which is known to inspire hard work in a person who has such choice available in his or her life (Terzi 2014; Waetjen 2006; Wilson-Strydom 2011).

Although the university encourages students with disabilities to disclose their disabilities so that the university can respond to their needs (NUL 2015), putting this policy into practice has met challenges. Information provided by the students is captured, but admissions are processed without using the data, thus forcing students with disabilities to compete for admission space equally with non-disabled peers. This makes the admission process at the institution unjust for students with disabilities. Access can be facilitated if disability data are used at the admission and planning stages. In this study, we argue, as do Leathwood (2005) and Troyna and Vincent (1995), that giving the same treatment to students with disabilities as we do for non-disabled students is not equitable. Equity is brought by efforts to compensate social disadvantage of students with disabilities (Dworkin 1981). Participation of students with disabilities is low because of, among other factors, the failure to make good use of disability data during admission. Withholding disability data has negatively influenced the quality of support from lecturers resulting in a reluctance to address, and the denial of, students’ needs.

Failure to utilise disability data can be attributed to poor planning and consultation between different university units and departments responsible for students’ support. As exemplified above, it took 7 weeks for a deaf student to have a sign language interpreter assigned to her; Lerato has mobility challenges, but her classes are set in lecture halls far apart so that she misses part of her lessons; and Raphael has to attend classes in storeyed buildings despite having mobility challenges. Essentially, students should be consulted about their needs for meaningful access (Claiborne et al. 2011), but unprocessed disability data result in exclusion. Furthermore, consistent with CHE’s (2012) finding, there were students with disabilities at institution whom the university did not disclose as disabled.

Equitable access is also negatively affected by the disability unit’s lack of development over the years. Although the unit started in 1999/2009, it has one SENA position and caters for one type of impairment. That is, support for students with disabilities is skewed towards students with visual impairments thus limiting support currently provided by the institution. Meanwhile, the support provided for students with visual impairments is also deficient: (1) students with visual impairment usually do not know timeously about services institution provides for them; (2) students described existing computers as obsolete and software outdated, an experience that was officially confirmed by SENA’s MEMO to the computer science unit; and (3) the library did not have books in e-text or Braille, therefore, the use of computers and Internet services was the only means of access to information for the students. Thus, students with visual impairments experienced more barriers than their peers with disabilities and non-disabled counterparts. Unlike their sighted peers who used library books, journals and other reference materials, students with visual impairments depended on lecture notes they recorded and material they downloaded from the Internet.

The institution lectures are offered face-to-face, and this study finds the practice unsuitable for students with disabilities. First, the university does not have sufficient resources such as projectors with the result that partially sighted students are forced to cope with lecturers’ handwriting. Lessons are fast-paced, and some students are not able to take notes. Access to curricula in this context is also denied because of a lack of prescribed books, hence overdependence on lectures. Students who fail to copy notes and students with visual impairments are not compensated (Dworkin 1981; Gewirtz 1998; Rawls 1971) with soft or hard copy notes. In addition, the institution does not have a policy which mandates lecturers to accommodate student diversity with the result that some lecturers are either indifferent or intolerant to students’ needs. For example, Motse is reluctant to ask for support because some lecturers respond negatively. Although one might hope that lecturers would accommodate students of their own free will, and in lieu of such policy, this not being the reality, some policy framework is necessary. To this end, Salmi and Bassett (2014) state that access is denied when opportunities are insufficient to enable students with disabilities to succeed in their chosen programmes. Students’ experiences show a lack of positive discrimination (Gewirtz 1998) which would facilitate equity, although there is evidence that attempts in the direction of positive discrimination are clear from the existence of the SESU. In fact, teaching and learning practices at the institution do not meet the basic equality principle of fairness where all have access to the same learning material. Lecturers’ lack of commitment to support students with disabilities may also be explained by their limited understanding of how to support students living with various disabilities.

Findings of the study further indicate that students with disabilities incur costs that their non-disabled peers are spared. They depend on technology to access information and so buy essential hardware such as tape-recorders. Our study found that when technology is used, but lecturers do not augment their teaching approaches accordingly, students cannot fully benefit from it. Students’ experiences reveal that they felt that they could not influence how their lecturers project their voices or move around the lecture rooms, and nor could they record lesson content successfully.

The university could enhance access to curricula if additional time for tests and examinations were provided equitably. Accommodations given to students with different disabilities are unsatisfactory to some. Consultations between students and staff could, when enabled, address issues surrounding students’ learning experiences. As Claiborne et al. (2011) observe, consultations with students living with disabilities give them an opportunity to define their needs. For example, Karabo is happy with the time concessions the university gives him but both Norma and Thomas do not understand how additional time in their tests and examinations was agreed upon as it is too limited. Thus, the university fails to respond to the needs of students with disabilities as promised (NUL 2015).

The teaching–learning facilitation at the HEI renders students with disabilities dependent. Students with mobility challenges arrive late for classes; those who are physically challenged and cannot copy notes are dependent on peers; and the partially sighted experience similar challenges resulting either from the pace of lessons or lecturers’ illegible handwriting. Students with visual impairments also depend on lecturers’ verbal and visual presentations. While Karabo says he only learns certain content in discussions leading to examinations, Motse says peers are not as clear in explaining concepts as the lecturers themselves. Therefore, students are left to catch up on content missed during class during uncoordinated discussions with friends. This leaves students with disabilities not empowered to study on their own and vulnerable to misinterpretation of the content. These students’ experiences demonstrate a lack of access to curricula and equity in the university teaching and learning practices.

## Ethical considerations

An ethical clearance procedure was followed with the University of South Africa before data were collected, and a clearance certificate was awarded to show that the study met basic ethical standards and posed no threat to the well-being of the participants. Permission to conduct the study was sought with the relevant officials of the National University of Lesotho, and all participants gave informed consent in writing and agreed to have their voices recorded. Participants were guaranteed that their identity would be kept confidential by using pseudonyms in transcription and reports. Only the primary researcher, as he did the transcriptions, had access to participants’ voice-recordings.

## Limitations of the study

The study has revealed that students with disabilities are not always identified, and disability information is not optimally utilised. Consequently, this study was not able to establish the total number of students with disabilities, their retention and graduation rates. Additionally, this study only identified lecturers who, at the time of data collection, either taught or were year tutors of the 11 student participants. Thus, there are other lecturers whose views are not included but may have taught and supported students with disabilities at the university.

## Conclusion and recommendations

This study’s findings offer insights beyond those of Matlosa and Matobo’s (2007) study which highlighted a lack of training for lecturers and insufficient technology, specifically that there was one computer with Internet access in the laboratory for students with visual impairment. We have identified which teaching and learning practices deny students access to learning spaces, and educational attainment, which they desire. We have noted that when disability data are not used profitably, it affects many aspects of access to education such as planning, providing positive discrimination and provision of requisite resources. We have also revealed that support mechanisms such as time concessions in tests and examinations may promote inequity despite being examples of practices that promote access.

The study concludes that students with disabilities are admissible, but there are restrictions for students with visual impairments. As access is not limited to entering the university, the university needs to do more to enhance opportunities for students with disabilities to succeed in their studies. Such efforts could usefully include a policy on how disability data should be used, and there is currently no leadership, from any department of the university, on how access to the university, its learning spaces and resources, should be provided. The institution only provides acceptable support for students with visual impairment; however, this support is also deficient. Students with disabilities have to facilitate their academic survival within the university programmes which ignore their needs.

It is therefore recommended that the HEI should pronounce itself regarding the practical implementation of regulations pertaining to students with disabilities. Specifically, we recommend that a clear policy on support of students with disabilities be developed by universities, with the aids of disabled persons organisations, students, and other key stakeholders. The policy should (1) outline how disability information should be used, (2) describe how different university departments must facilitate students’ support, (3) outline how teaching and learning resources must be enhanced to facilitate access, (4) stimulate research on the success and completion rates of students with disabilities the university enrols and (5) explore ways to adapt programmes currently inaccessible to students with disabilities. Although having such a policy would not guarantee the full inclusion and effective participation of students with disabilities in HEIs, it would be a valuable initial step by providing a reference point and framework for both students and institutions to begin to redress the current imbalances in access.
